# Small bowel cancer arising from a scar site more than 60 years after ileostomy closure: a case report

**DOI:** 10.1093/jscr/rjac485

**Published:** 2022-10-31

**Authors:** Akihiro Kohata, Masatoshi Kochi, Nao Kitasaki, Tomoyuki Abe, Ryuichi Hotta, Masashi Inoue, Takuya Hattori, Kazuhiro Toyota, Tadateru Takahashi

**Affiliations:** Department of Surgery, National Hospital Organization Higashi Hiroshima Medical Center, Higashihiroshima, Japan; Department of Surgery, National Hospital Organization Higashi Hiroshima Medical Center, Higashihiroshima, Japan; Department of Surgery, National Hospital Organization Higashi Hiroshima Medical Center, Higashihiroshima, Japan; Department of Surgery, National Hospital Organization Higashi Hiroshima Medical Center, Higashihiroshima, Japan; Department of Surgery, National Hospital Organization Higashi Hiroshima Medical Center, Higashihiroshima, Japan; Department of Surgery, National Hospital Organization Higashi Hiroshima Medical Center, Higashihiroshima, Japan; Department of Pathology, National Hospital Organization Higashi Hiroshima Medical Center, Higashihiroshima, Japan; Department of Surgery, National Hospital Organization Higashi Hiroshima Medical Center, Higashihiroshima, Japan; Department of Surgery, National Hospital Organization Higashi Hiroshima Medical Center, Higashihiroshima, Japan

## Abstract

Although small bowel cancer is rare, cases of carcinoma arising from the abdominal wall have not been reported. We report a case of a tumor arising from a stoma scar site, following ileostomy closure that was performed 60 years earlier. The tumor was resected for both therapeutic and diagnostic purposes and was found to be a primary cancer of the small intestine. The small intestinal mucosa survived long-term at the stoma scar site and developed carcinoma. No similar reports of small bowel cancer arising from the mucosa at the stoma scar site (on the abdominal wall) exist. After tumor resection, the patient received chemotherapy for lung metastases and has survived, thus far, for 2 years since the surgery.

## INTRODUCTION

Small bowel cancer is one of the rarest primary tumors of the intestinal tract. Small bowel cancer occurs in the small intestine of the abdominal cavity and, in some cases, in the ileostomy of patients after colorectal resection for inflammatory bowel disease (IBD).

The liver is the most common metastatic site for small bowel cancer, followed by the peritoneum and distant lymph nodes [1]. Colorectal cancer may also metastasize to the port site after laparoscopic surgery [2], where this metastasis is thought to arise from the seeding of cancer cells during laparoscopic surgery. Although case reports of small bowel cancer metastasizing to the abdominal wall exist [3], there are no existing reports of carcinoma arising from the abdominal wall.

We encountered a case of small bowel cancer arising from the abdominal wall at an ileostomy scar site >60 years after ileostomy closure. We consider this an extremely rare case of small bowel cancer arising from the abdominal wall.

## CASE REPORT

**Figure 1:**
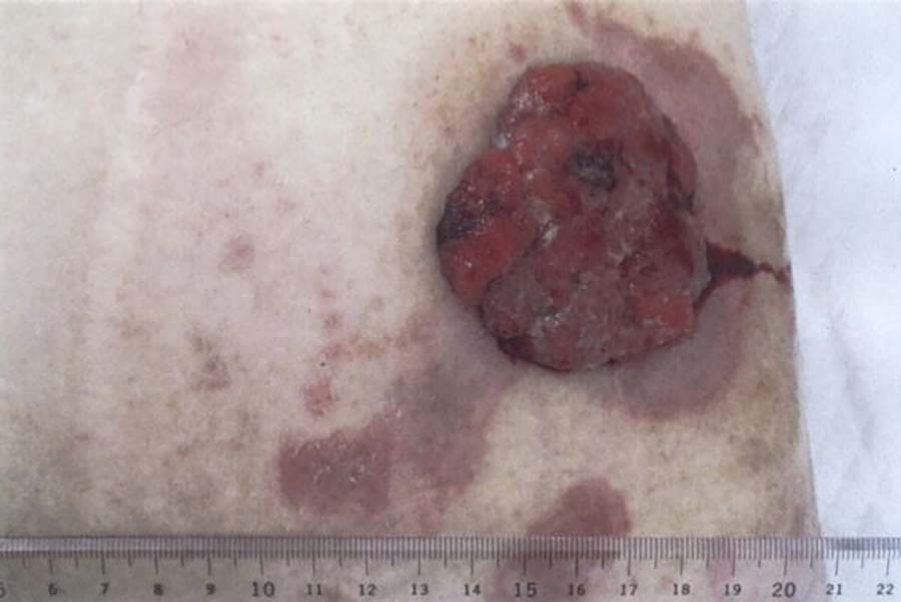
The mass was hemorrhagic, and protruded from the stoma scar.

A 73-year-old man presented to our hospital with the chief complaint of a mass in the abdominal wall. The mass was reddish-brown, firm, hemorrhagic and protruded from the stoma scar with multiple nodules (Fig. 1). He underwent surgery for intestinal volvulus when he was 6 years old, and ileostomy was performed thereafter due to postoperative suture failure. The ileostomy was then closed. The abdominal wall mass was located at the ileostomy scar site.

**Figure 2:**
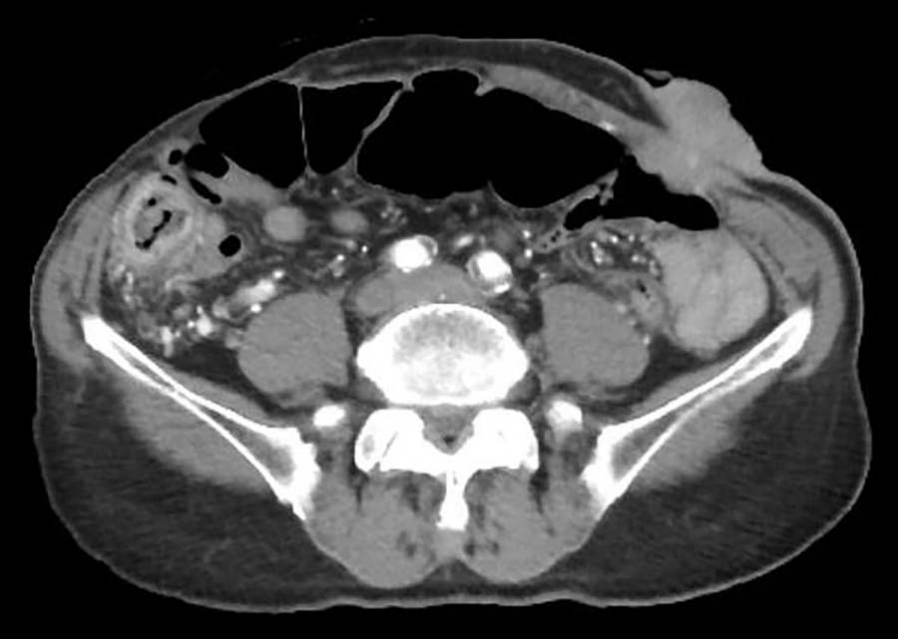
Computed tomography showed irregular mass at the abdominal wall.

**Figure 3:**
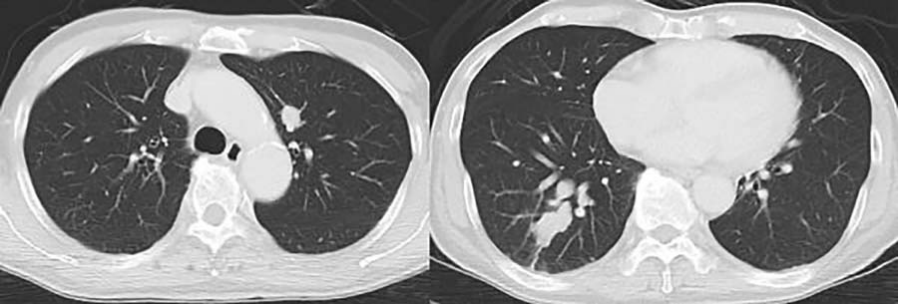
Computed tomography also showed irregular lung masses.

Contrast-enhanced computed tomography revealed a giant irregular mass (45 × 35 mm) in the abdominal wall (Fig. 2). No other intra-abdominal neoplasm were present; however, an irregular lung mass measuring 50 mm in size in the S10 region of the right lobe and a mass measuring 12 mm in size in the S1/2 region of the left lobe were found (Fig. 3). Total gastrointestinal endoscopy, including capsule endoscopy, revealed no neoplasm in the intestinal tract (Fig. 4a–c).

**Figure 4a:**
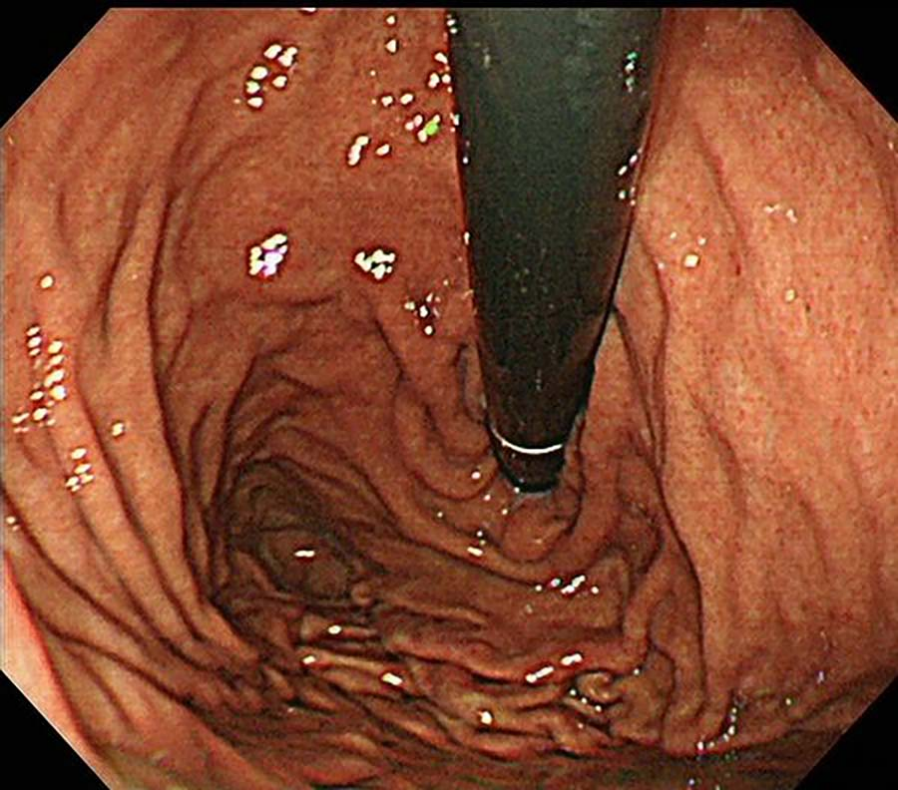
Upper gastrointestinal endoscopy revealed no neoplasm.

**Figure 4b:**
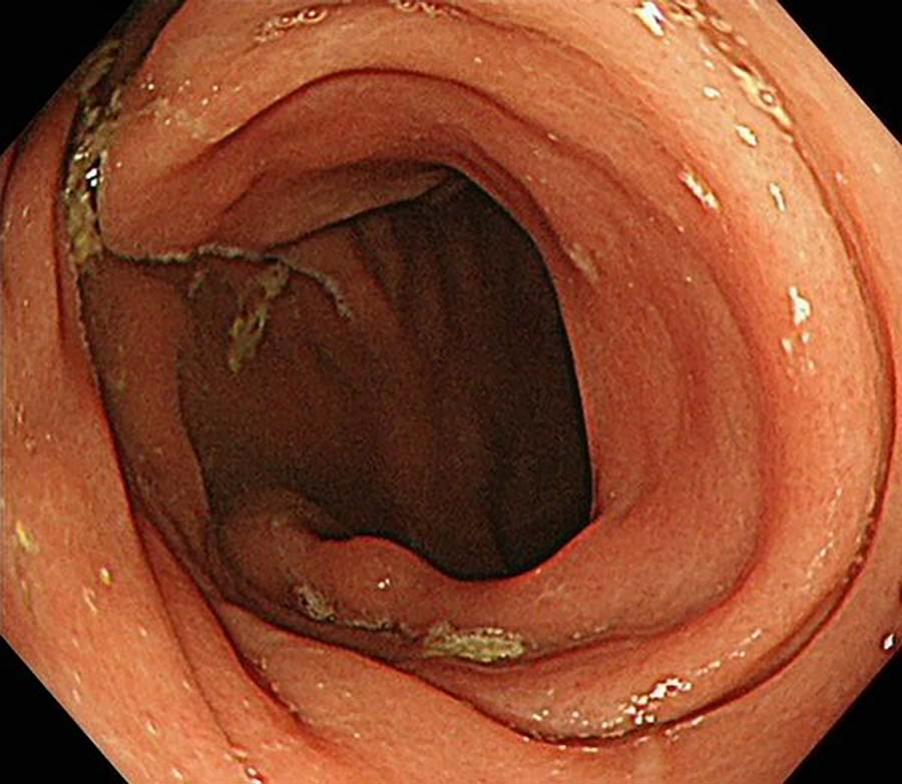
Colonoscopy also revealed no neoplasm.

**Figure 4c:**
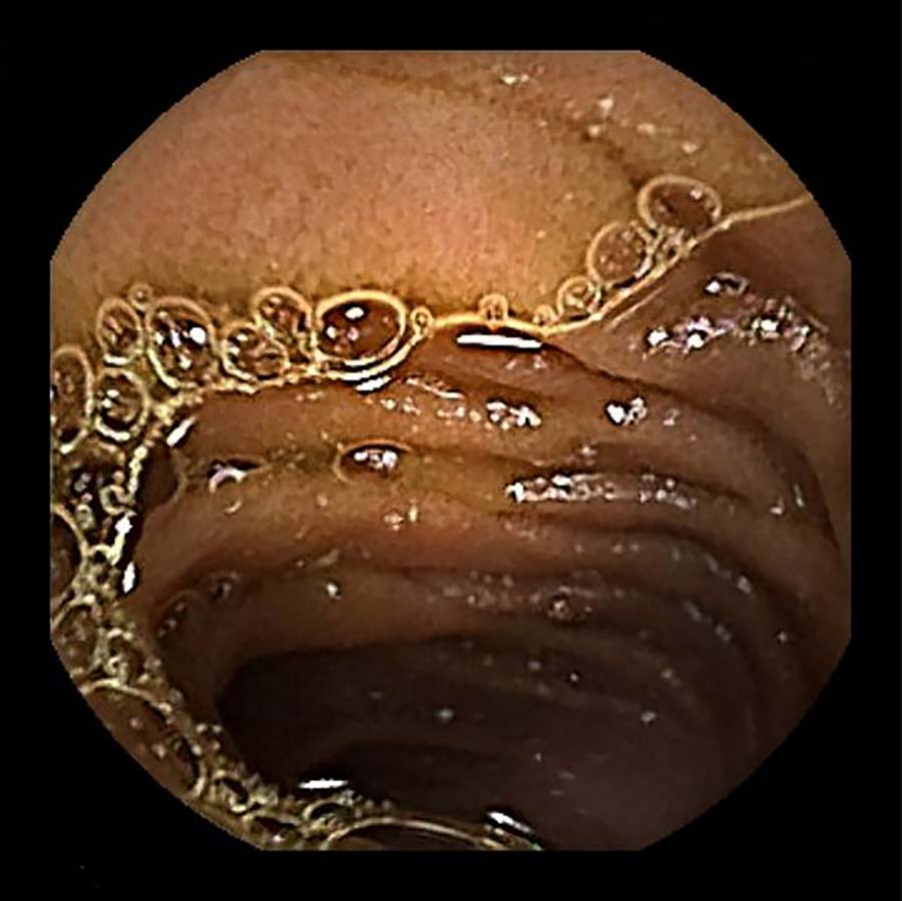
Capsule endoscopy also revealed no neoplasm.

**Figure 5:**
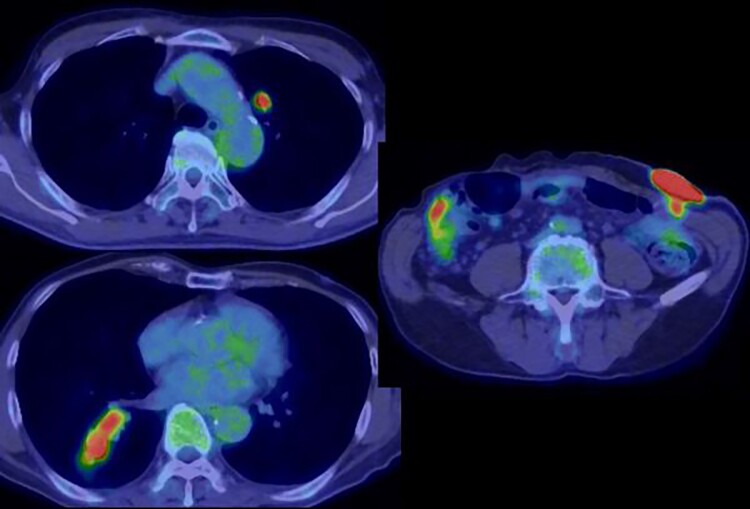
Positron emission tomography revealed an abnormal accumulation of a mass in the abdominal wall and bilateral lung.

**Figure 6:**
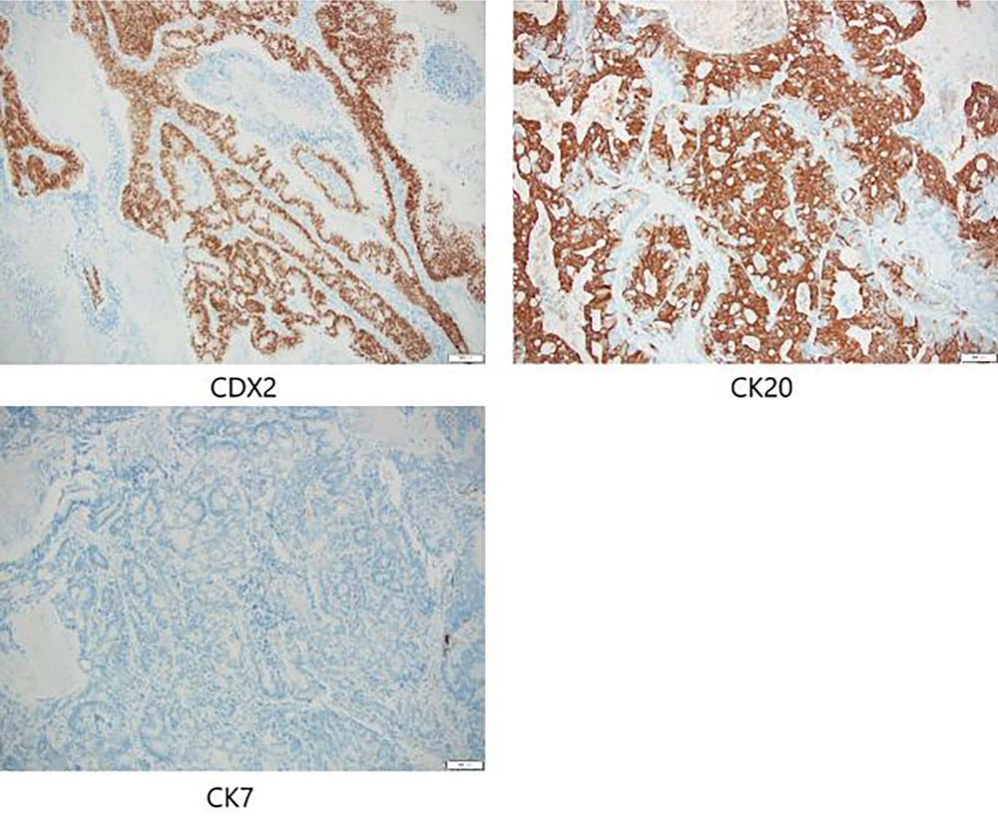
Immunostaining of biopsy specimen was strongly positive for CDX2/CK20 and negative for CK7.

Positron emission tomography revealed abnormal accumulation of a mass in the left abdominal wall (with a maximum standardized uptake value [SUVmax] of 38.7) and slight accumulation of a mass in the S10 (SUVmax 5.7) and S1/2 (SUVmax 5.5) regions (Fig. 5), but no abnormal accumulation in the abdominal cavity was found. Biopsy of the abdominal wall mass revealed highly- to moderately-differentiated ductal adenocarcinoma cells. Immunostaining was strongly positive for CDX2/CK20 and negative for CK7 (Fig. 6), suggesting adenocarcinoma of intestinal origin. Transbronchial biopsy of the lung mass was also positive for CDX2/CK20 and negative for CK7/TTF-1/napsin A. The tumor cells had an eosinophilic cytoplasm and small nuclei of unequal sizes, similar to the histology of the biopsy specimen of the previously described abdominal wall mass.

Since the tumor was causing pain, and the bleeding from the tumor interfered with the patient’s activities of daily living, we decided to resect the tumor. The tumor was resected under general anesthesia. A defect in the rectus abdominis muscle of the abdominal wall was repaired using mesh. The pathological findings of the resected tumor were similar to those of the previously described mass and to the lung biopsy specimens, and the tumor was confirmed to be an adenocarcinoma of intestinal origin. Since no primary tumor was present in the intestinal tract, the tumor was thought to have originated from the small intestinal tissue remaining in the ileostomy scar, which then metastasized to the lungs.

The patient underwent postoperative chemotherapy and is still alive 2 years after surgery.

## DISCUSSION

The diagnosis of malignant tumors arising in the abdominal wall includes primary soft tissue tumors of the abdominal wall and abdominal wall metastasis of cancer from other organs.

In the present case, the tumor on the patient’s abdominal wall was a lesion continuous from the deep to superficial layers of the abdominal wall. Based on immunostaining results, we diagnosed the patient with intestinal-type adenocarcinoma. Hiramatsu *et al.* [4] stated that when cancer and ectopic tissue coexist, identifying the transition zone between them is critical in determining the origin of the cancer. In this patient, non-neoplastic epithelial cells were present adjacent to neoplastic epithelial cells (Fig. 7a–c). These non-neoplastic epithelial cells were CDX2/CK20 positive and CK7 negative, indicating that the tumor comprised ectopic tissue of intestinal origin.

**Figure 7a:**
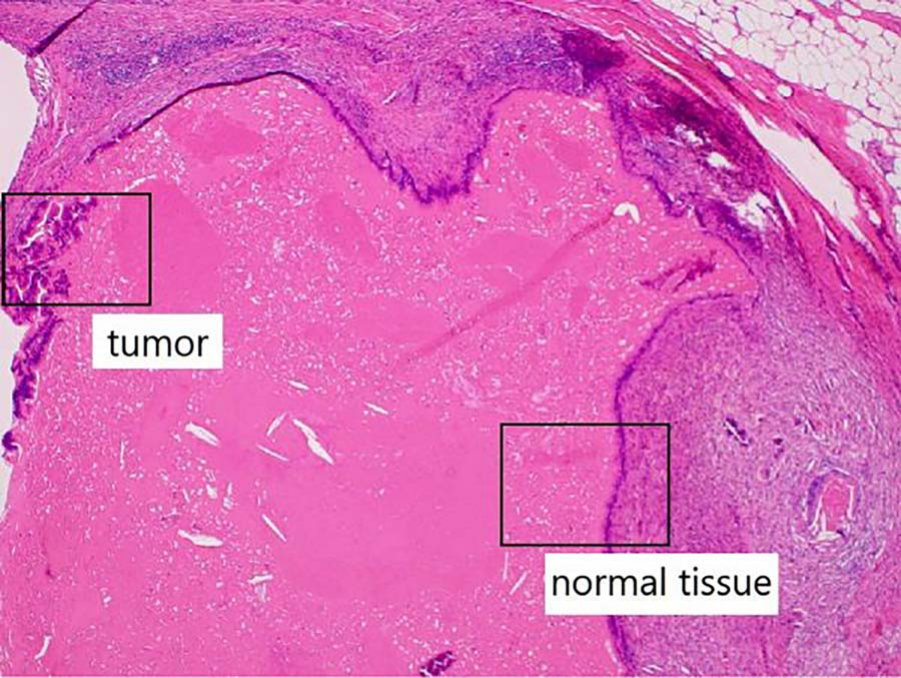
Non-neoplastic epithelial cells were present adjacent to neoplastic epithelial cells.

**Figure 7b:**
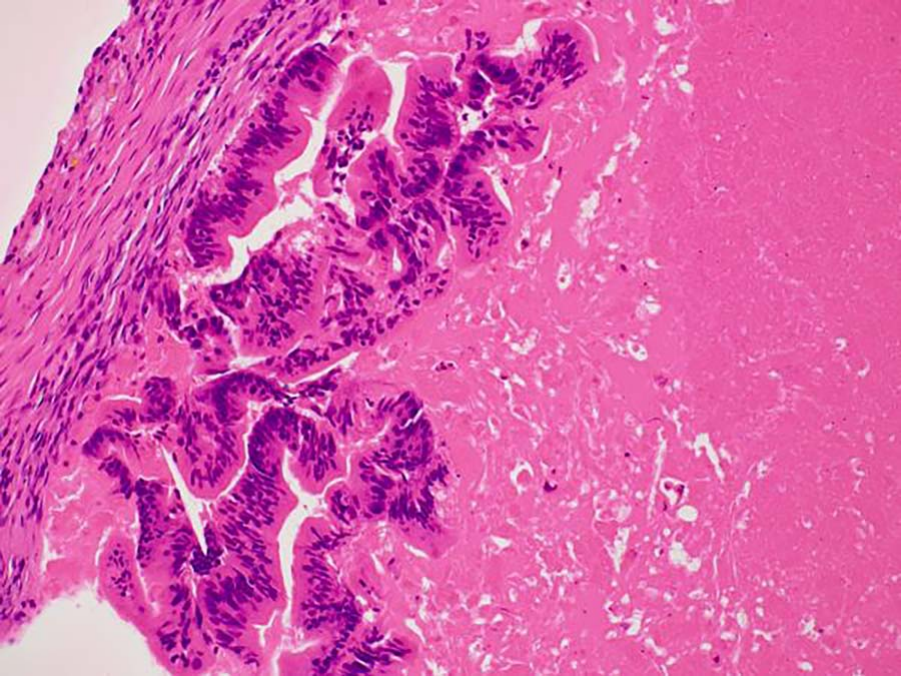
Differentiated ductal adenocarcinoma grew irregularly in a gourd-like pattern at the tumor area.

**Figure 7c:**
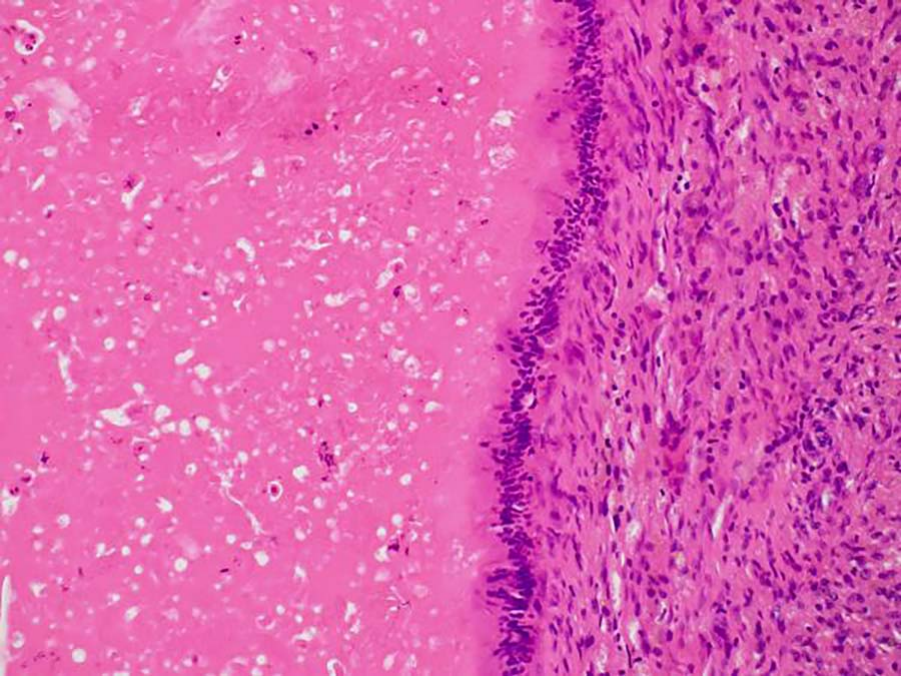
Non-neoplastic intestinal epithelium remained at the normal tissue area.

Reports of carcinogenesis from ileostomies exist; however, in many cases, IBD is a background disease. Metzger *et al*. [5] suggested that colonic metaplasia due to chronic inflammation of the mucocutaneous junction may be the cause of carcinogenesis in ileostomies. Hammad *et al*. [6] described the malignant transformation of pre-existing adenomas as a cause of carcinogenesis in ileostomies. These researchers also reported that many other factors (including colonic metaplasia and dysplasia of the ileal mucosa, chronic irritation due to physical or chemical trauma and ileitis) are involved in this process. Considering our patient’s history, it is assumed that the normal small intestinal mucosa grew on the ileostomy scar, survived for a long period of time, and then developed into carcinoma.

To the best of our knowledge, no reports of small bowel carcinogenesis from ileostomy scars exist. In addition, it is unclear why the small intestinal mucosa could survive for such a long period at the ileostomy scar site. To investigate the reasons, we focused on the wound-healing process during stoma closure surgery. Skin wounds heal through processes such as inflammation, granulation tissue formation, angiogenesis, re-epithelialization and remodeling. These processes are regulated by interactions between different cell types (including keratinocytes, fibroblasts, endothelial cells and inflammatory cells) that migrate to the wound site. These interactions are also mediated by intrinsic factors (such as cytokines) that are secreted by these cells [7]. We believe that among other effects, the angiogenic effect of intrinsic factors may have created a favorable environment in the stoma scar area for the ectopic small intestinal mucosa to survive.

In conclusion, this is the first case of small bowel cancer arising from the abdominal wall. Therefore, when a tumor growing on the abdominal wall in the ileostomy scar area is detected, the possibility of small bowel cancer should be kept in mind during examination.
